# Supplementary material for: Modeling single-cell phenotypes links yeast stress acclimation to transcriptional repression and pre-stress cellular states
Source: eLife. 2022 Nov 9;11:e82017. doi: 10.7554/eLife.82017 (PMC9678356; doi:10.7554/eLife.82017)
Supplement: Supplementary file 1. [file elife-82017-supp1.docx]

**Supplementary File 1. Permutations of coordinately timed peaks in cells in two-cell colonies**

|  | number of colonies with coordinated peaks  (out of 56 total) | Fraction of permutations with coordinated peak number equal or greater to actual number of colonies with coordinated peaks* |
| --- | --- | --- |
| Dot6 coordinated peak | 15 | 9.5 x 10^-4^ |
| Msn2 coordinated peak | 6 | 0.19 |

* There were 50,000 iterations of permutations for each comparison.
