# Supplementary material for: Modeling single-cell phenotypes links yeast stress acclimation to transcriptional repression and pre-stress cellular states
Source: eLife. 2022 Nov 9;11:e82017. doi: 10.7554/eLife.82017 (PMC9678356; doi:10.7554/eLife.82017)
Supplement: Supplementary file 2. — The number of cells in each mclust cluster from Figure 4 is shown along with the number of those cells from each of three biological replicates. P-values from binomial probability tests (see Methods) are shown and those significant after Holm-Bonferroni correction (namely Cluster 9 which was enriched for cells from replicate 3) are indicated with an asterisk. [file elife-82017-supp2.docx]

**Supplementary File 2. Cell subpopulations are identified in multiple biological replicates**

|  |  | Cell number from each biological replicate | | | P-value (before Holm-Bonferroni correction) | | |
| --- | --- | --- | --- | --- | --- | --- | --- |
| mclust cluster | Number of cells in cluster | Biological replicate 1 | Biological replicate 2 | Biological replicate 3 | Biological replicate 1 | Biological replicate 2 | Biological replicate 3 |
| 1 | 61 | 23 | 21 | 17 | 0.46 | 0.13 | 0.94 |
| 2 | 9 | 3 | 3 | 3 | 0.69 | 0.46 | 0.69 |
| 3 | 18 | 7 | 9 | 2 | 0.50 | 0.034 | 1.00 |
| 4 | 13 | 3 | 7 | 3 | 0.90 | 0.038 | 0.90 |
| 5 | 22 | 10 | 4 | 8 | 0.25 | 0.89 | 0.58 |
| 6 | 16 | 3 | 2 | 11 | 0.96 | 0.96 | 0.0086 |
| 7 | 20 | 5 | 3 | 12 | 0.90 | 0.94 | 0.027 |
| 8 | 5 | 1 | 0 | 4 | 0.90 | 1.00 | 0.062 |
| 9 | 16 | 3 | 0 | 13 | 0.96 | 1.00 | 0.0003* |
| 10 | 6 | 4 | 1 | 1 | 0.13 | 0.85 | 0.93 |
| 11 | 35 | 17 | 11 | 7 | 0.094 | 0.35 | 0.99 |

*Significant after Holm-Bonferroni correction.

The number of cells in each mclust cluster from Figure 4 is shown along with the number of those cells from each of three biological replicates. P-values from binomial probability tests (see Methods) are shown and those significant after Holm-Bonferroni correction (namely Cluster 9 which was enriched for cells from replicate 3) are indicated with an asterisk.
