# Supplementary material for: Modeling single-cell phenotypes links yeast stress acclimation to transcriptional repression and pre-stress cellular states
Source: eLife. 2022 Nov 9;11:e82017. doi: 10.7554/eLife.82017 (PMC9678356; doi:10.7554/eLife.82017)
Supplement: Supplementary file 3. [file elife-82017-supp3.docx]

**Supplementary File 3. Multiple Linear Models: variables, significance, and explained variance.**

|  | Multiple Linear Model  (p-values of included variables) | | | | | |
| --- | --- | --- | --- | --- | --- | --- |
| Model Parameter | Model 1 | Model 2** | | Model 3*** | Model 4 | Model 5 |
| Intercept | 3.7E-04 | | 8.9E-07 | 3.1E-05 | 0.0009 | 1.75E-05 |
| Dot6 pre-stress AUC | 0.016 | | 0.0004 | 0.00011 | 0.0070 |  |
| Msn2 pre-stress AUC | NaN | |  |  |  |  |
| Msn2 – Dot6 AUC | 0.468 | |  |  |  |  |
| Sum of Dot6 peak heights (pre-stress) | 0.767 | |  |  |  |  |
| Sum of Msn2 peak heights  (pre-stress) | 0.016 | | 0.0024 | 0.00010 | 0.0007 |  |
| Dot6 acute stress peak height | 1.4E-07 | | 6.3E-10 | 1.0E-08 | 3.41E-10 | 0.00013 |
| Msn2 acute stress peak height | 0.125 | |  |  |  |  |
| Msn2 acclimation AUC | 0.017 | | 0.0084 |  |  |  |
| G1 (at time of stress)* | 0.438 | |  |  |  |  |
| S-phase (at time of stress) | 0.528 | |  |  |  |  |
| G2 (at time of stress) | 0.233 | |  |  |  |  |
| M-phase (at time of stress) | 0.221 | |  |  |  |  |
| Cell/colony size at experiment start | 0.042 | | 0.0104 |  |  |  |
| Pre-stress growth rate | 2.2E-06 | | 4.0E-07 | 2.7E-08 |  | 1.59E-06 |
|  |  | |  |  |  |  |
| R^2^ (explained variance) | 0.45 | | 0.40 | 0.35 | 0.22 | 0.26 |

*Cell-cycle phase included four binary parameters (i.e. dummy variables) corresponding to the four cell-cycle phases (G1, S, G2 and M phase). See the Methods section for how these were scored.

** Model 2 only included all the significant dependent variables from Model 1.

*** Model 3 excluded Msn2 acclimation AUC and Cell/colony size from Model 2 as the p-values did not pass Holm-Bonferroni correction.
