# Supplementary material for: Modeling single-cell phenotypes links yeast stress acclimation to transcriptional repression and pre-stress cellular states
Source: eLife. 2022 Nov 9;11:e82017. doi: 10.7554/eLife.82017 (PMC9678356; doi:10.7554/eLife.82017)
Supplement: MDAR checklist [file elife-82017-mdarchecklist1.pdf]

Westbrook Centre
Milton Road
Cambridge CB4 1YG, UK
P 01223 855340
W elifesciences.org
T @elife

Materials Design Analysis Reporting (MDAR) Checklist for Authors

The MDAR framework establishes a minimum set of requirements in transparent reporting mainly applicable to studies in the life sciences.

eLife asks authors to **provide detailed information within their article** to facilitate the interpretation and replication of their work. Authors can also upload supporting materials to comply with relevant reporting guidelines for health-related research (see EQUATOR Network), life science research (see the BioSharing Information Resource), or animal research (see the ARRIVE Guidelines and the STRANGE Framework; for details, see *eLife*'s Journal Policies). Where applicable, authors should refer to any relevant reporting standards materials in this form.

For all that apply, please note **where in the article** the information is provided. Please note that we also collect information about data availability and ethics in the submission form.

Materials:

Newly created materials	Indicate where provided: section/figure legend	N/A
The manuscript includes a dedicated "materials availability statement" providing transparent disclosure about availability of newly created materials including details on how materials can be accessed and describing any restrictions on access.	Statement within Methods includes this information: “strains available upon request”.	

Antibodies	Indicate where provided: section/figure legend	N/A
For commercial reagents, provide supplier name, catalogue number and RRID, if available.		N/A

DNA and RNA sequences	Indicate where provided: section/figure legend	N/A
Short novel DNA or RNA including primers, probes: Sequences should be included or deposited in a public repository.		N/A

Cell materials	Indicate where provided: section/figure legend	N/A
Cell lines: Provide species information, strain. Provide accession number in repository OR supplier name, catalog number, clone number, OR RRID.		N/A
Primary cultures: Provide species, strain, sex of origin, genetic modification status.		N/A

**Experimental animals**	**Indicate where provided: section/figure legend**	**N/A**
Laboratory animals or Model organisms: Provide species, strain, sex, age, genetic modification status. Provide accession number in repository OR supplier name, catalog number, clone number, OR RRID.		N/A
Animal observed in or captured from the field: Provide species, sex, and age where possible.		N/A

**Plants and microbes**	**Indicate where provided: section/figure legend**	**N/A**
Plants: provide species and strain, ecotype and cultivar where relevant, unique accession number if available, and source (including location for collected wild specimens).		N/A
Microbes: provide species and strain, unique accession number if available, and source.	Specific strains used are described in Methods. They are:<br>- AGY1328: *Saccharomyces cerevisiae*; BY4741; *DOT6-GFP(S65T)-His3MX, MSN2-mCherry-HYGMX;* Source: Gasch et al., 2017<br>- AGY1813: *Saccharomyces cerevisiae*; BY4741; *DOT6-GFP(S65T)-His3MX, MSN2-mCherry-HYGMX, CTT1-iRFP-KanMX*; Source: this study<br>- AGY1363: *Saccharomyces cerevisiae*; BY4741; *dot6::KAN tod6::HYG CTT1-GFP(S65T)-His3MX;* Source: Ho et al., 2018	

**Human research participants**	**Indicate where provided: section/figure legend) or state if these demographics were not collected**	**N/A**
If collected and within the bounds of privacy constraints report on age, sex, gender and ethnicity for all study participants.		N/A

Design:

**Study protocol**	**Indicate where provided: section/figure legend**	**N/A**

If the study protocol has been pre-registered, provide DOI. For clinical trials, provide the trial registration number OR cite DOI.		N/A

**Laboratory protocol**	**Indicate where provided: section/figure legend**	**N/A**
Provide DOI OR other citation details if detailed step-by-step protocols are available.		N/A

**Experimental study design (statistics details) ***		
**For in vivo studies: State whether and how the following have been done**	**Indicate where provided: section/figure legend. If it could have been done, but was not, write “not done”**	**N/A**
Sample size determination	Sample size was determined based on statistical power determined during the analysis. Sample sizes are stated both in the Results and Methods sections. We established strict guidelines for quality control and collected enough cells to ensure that we had at least 200 cells from three replicates (from strain AGY1328) that met high-quality metrics (in Methods). We used similar data collection and identical analysis metrics on three additional replicates of a similar strain (AGY1813), and also analyzed over 200 cells from these replicates.	
Randomisation	Where relevant, subsets of cells were analyzed to ensure that results were not biased by the dataset. Statistical tests were applied to ensure that results were reproduced across all three biological replicates.	
Blinding		N/A
Inclusion/exclusion criteria	Specific inclusions/exclusion criteria are stated in the Methods section, including quality control filters. The number of cells included and excluded due to these filters are also listed.	

Sample definition and in-laboratory replication	Indicate where provided: section/figure legend	N/A
State number of times the experiment was replicated in the laboratory.	Found in Methods. Three biological replicates (separate experimental/batch runs) were used to acquire data (strain AGY1328). Three additional biological replicates from a similar strain (AGY1813) were used to further validate and expound upon initially reported results.	
Define whether data describe technical or biological replicates.	Llisted in Results and Methods. Data describe biological replicates. A biological replicate is defined as an experimental microscopy run performed on a separate day.	

Ethics	Indicate where provided: section/submission form	N/A
Studies involving human participants: State details of authority granting ethics approval (IRB or equivalent committee(s), provide reference number for approval.		N/A
Studies involving experimental animals: State details of authority granting ethics approval (IRB or equivalent committee(s), provide reference number for approval.		N/A
Studies involving specimen and field samples: State if relevant permits obtained, provide details of authority approving study; if none were required, explain why.		N/A

Dual Use Research of Concern (DURC)	Indicate where provided: section/submission form	N/A
If study is subject to dual use research of concern regulations, state the authority granting approval and reference number for the regulatory approval.		N/A

Analysis:

Attrition	Indicate where provided: section/figure legend	N/A

Describe whether exclusion criteria were pre-established. Report if sample or data points were omitted from analysis. If yes, report if this was due to attrition or intentional exclusion and provide justification.	Found in Methods. Exclusion criteria was established during this study. Cells were omitted from analysis if they did not meet certain quality control guidelines (see Methods for specific guidelines and justifications).	

**Statistics**	**Indicate where provided: section/figure legend**	**N/A**
Describe statistical tests used and justify choice of tests.	- mclust: (Methods and Figure 4, Figure 4 – supplement 1 legend, Table S2)<br>- Binomial probabilities: used to determine if each cluster contained more cells from one of the three biological replicates than expected by change (Methods)<br>- Holm-Bonferroni: used for multiple hypothesis correction (Methods)<br>- Permutations: used to identify if matched peaks of Msn2 and Dot6 and coordinated peaks between two-cell colonies were more coordinated than expected by chance (Methods and Table S1)<br>- Wilcoxon Rank Sum test: used to identify differences in growth rates among cell clusters (Figure 1, Figure 5 – supplement 1)<br>- Linear modeling: (Methods and Figure 5, Figure 5 – supplement 1, Figure 6, Figure 6- supplement 1, Figure 6 – supplement 2, Figure 7, Figure 7 – supplement 1, Table S3<br>- Principle component regression: (Methods and Figure 6,	

**Data availability**	**Indicate where provided: section/submission form**	**N/A**
For newly created and reused datasets, the manuscript includes a data availability statement that provides details for access (or notes restrictions on access).	Data were newly created. Raw data used in analyses are included in supplement files (Bergen-Kocik_AGY1328_Single_Cell_Measurments_Source Data and Legend and Bergen_Kocik_AGY1813_Single_Cell_Measurements Source Data and Legend).	
When newly created datasets are publicly available, provide accession number in repository OR DOI and licensing details where available.		N/A

If reused data is publicly available provide accession number in repository OR DOI, OR URL, OR citation.		N/A

**Code availability**	**Indicate where provided: section/figure legend**	**N/A**
For any computer code/software/mathematical algorithms essential for replicating the main findings of the study, whether newly generated or re-used, the manuscript includes a data availability statement that provides details for access or notes restrictions.	MATLAB scripts were written for this manuscript. Relevant scripts for analyzing data are included in supplemental files (segment_find_cells.m and track_cells_data_extract.m)	
Where newly generated code is publicly available, provide accession number in repository, OR DOI OR URL and licensing details where available. State any restrictions on code availability or accessibility.		N/A
If reused code is publicly available provide accession number in repository OR DOI OR URL, OR citation.		N/A

Reporting:

The MDAR framework recommends adoption of discipline-specific guidelines, established and endorsed through community initiatives.

**Adherence to community standards**	**Indicate where provided: section/figure legend**	**N/A**
State if relevant guidelines (e.g., ICMJE, MIBBI, ARRIVE, STRANGE) have been followed, and whether a checklist (e.g., CONSORT, PRISMA, ARRIVE) is provided with the manuscript.		N/A

---

* We provide the following guidance regarding transparent reporting and statistics; we also refer authors to Ten common statistical mistakes to watch out for when writing or reviewing a manuscript.

Sample-size estimation

- You should state whether an appropriate sample size was computed when the study was being designed
- You should state the statistical method of sample size computation and any required assumptions
- If no explicit power analysis was used, you should describe how you decided what sample (replicate) size (number) to use

Replicates

- You should report how often each experiment was performed
- You should include a definition of biological versus technical replication
- The data obtained should be provided and sufficient information should be provided to indicate the

number of independent biological and/or technical replicates
- If you encountered any outliers, you should describe how these were handled
- Criteria for exclusion/inclusion of data should be clearly stated
- High-throughput sequence data should be uploaded before submission, with a private link for reviewers provided (these are available from both GEO and ArrayExpress)

Statistical reporting
- Statistical analysis methods should be described and justified
- Raw data should be presented in figures whenever informative to do so (typically when N per group is less than 10)
- For each experiment, you should identify the statistical tests used, exact values of N, definitions of center, methods of multiple test correction, and dispersion and precision measures (e.g., mean, median, SD, SEM, confidence intervals; and, for the major substantive results, a measure of effect size (e.g., Pearson's r, Cohen's d)
- Report exact p-values wherever possible alongside the summary statistics and 95% confidence intervals. These should be reported for all key questions and not only when the p-value is less than 0.05.

Group allocation
- Indicate how samples were allocated into experimental groups (in the case of clinical studies, please specify allocation to treatment method); if randomization was used, please also state if restricted randomization was applied
- Indicate if masking was used during group allocation, data collection and/or data analysis
